# Body Mass Index and Sociodemographic Predictors of School Lunch Purchase Behavior during a Year-Long Environmental Intervention in Middle School

**DOI:** 10.3390/bs5020324

**Published:** 2015-06-10

**Authors:** Jacey A. Greece, Alyssa Kratze, William DeJong, Yvette C. Cozier, Paula A. Quatromoni

**Affiliations:** 1 Department of Community Health Sciences, Boston University School of Public Health, 801 Massachusetts Avenue, 4th Floor, Boston, MA 02118, USA; E-Mails: akratze@bu.edu (A.K.); wdejong@bu.edu (W.D.); 2 Department of Epidemiology, Boston University School of Public Health, 715 Albany Street, 3rd Floor, Boston, MA 02118, USA; E-Mails: yvettec@bu.edu (Y.C.C.); paulaq@bu.edu (P.A.Q.); 3 Slone Epidemiology Center at Boston University, 1010 Commonwealth Avenue, Boston, MA 02215, USA; 4 Department of Health Sciences, Boston University Sargent College of Health and Rehabilitation Sciences, 635 Commonwealth Avenue, Boston, MA 02215, USA

**Keywords:** child nutrition, school foodservice, body mass index, environmental intervention, school lunch participation, nutrition policy

## Abstract

Modifying the school food environment is on the national agenda as one strategy to improve the nutritional quality of children’s diets. Because few environmental-level interventions have been rigorously evaluated, the evidence base to inform programs and policies is limited. Of concern is the impact that changes to cafeteria offerings will have on participation in school meal programs. This study evaluates school lunch participation in the setting of a year-long middle school cafeteria intervention by examining the association between body mass index (BMI), sociodemographics, and the purchases of school lunch meals. IMOVE meals were healthier choices that met stringent nutritional criteria and were offered alongside standard lunch meals. Students who were overweight had a significantly higher purchase rate for both types of meals compared to those with a healthy BMI. Non-white race, younger age, being male, and low-income status were also significantly associated with participation in school lunch. Results indicate that nutritionally vulnerable students participate in school lunch and are equally likely to buy healthy alternatives or standard meals. This behavioral observation has important implications for school foodservice programs and policies. These results are timely given recent federal legislation to improve the school food environment to influence students’ food choice behaviors.

## 1. Introduction

Forty percent of children attending United States public schools are overweight, with more than 20% classified as obese. Improving the school food environment and strengthening school wellness policies have been priorities both locally and nationally [1]. In 2010, the *Healthy, Hunger-Free Kids Act* (HHFKA) gave the United Stated Department of Agriculture (USDA) the authority to set nutritional standards for foods sold in schools, including the National School Lunch Program (NSLP), School Breakfast Program (SBP), and competitive foods sold at schools, and to provide reimbursement to schools that meet updated nutritional standards based on the 2010 Dietary Guidelines for Americans. Under this Act, the USDA is also empowered to set standards for nutrition education, physical activity, and wellness policies, and to establish networks between schools and local farms to create community gardens to ensure use of local produce in the school setting [2,3].

The HHFKA expanded the scope of nutritional standards in the school environment by applying the USDA’s regulatory authority to all foods offered either through or outside of the school meals program, including á la carte, vending machine, and other foods sold in school settings [4]. The HHFKA has been criticized, however, for creating healthier meals that cost more, are less appetizing to students, and have smaller portions and therefore may be inadequate for some students due to the calorie restrictions imposed by the new standards [5]. In 2012–2013, concerns about HHFKA’s implementation arose in 48 of the 50 U.S. states. Reports indicated that compared to the year before, nearly one million children (3.7%) no longer participated in the school lunch program due to the changes that occurred [6]. In a more recent study, however, student acceptance of the new school lunches was perceived by elementary school personnel to be “reasonable” [7]. More rigorous research is needed to better understand both the acceptability and nutritional impact of the changes being made in the school food environment.

The NSLP and competitive foods offered in school can potentially impact the eating behaviors and health status of students. Studies have reached different conclusions about the impact of NSLP participation on children’s body weight, most likely due to different research methods. One study showed no effect of the NSLP on children’s weight [1] while other investigations have suggested that the program could be contributing to the childhood obesity epidemic [8,9]. For example, one study showed that participation in the NSLP prior to the implementation of the HHFKA regulations increased the probability of being obese due to the excess calories present in the standard school lunch [10]. Another study found that while NSLP participation increased the intake of health-promoting nutrients, it also contributed to higher intake levels of fat [11]. At the same time, another study showed that children were at increased risk of gaining weight during the summer months due to the absence of the school meal program and greater consumption of food at home [12]. As evident, school meals programs play a prominent role in protecting the health of students by providing nutritious meals to socioeconomically disadvantaged children. 

A cross-sectional study of a nationally representative sample of students grades 1 through 12 conducted prior to the passing of HHFKA found no association between school lunch participation and either higher BMI or higher incidence of overweight or obesity [1]. A quasi-experimental study conducted after the passing of HHFKA examined a sample of 8th grade students across 40 states to assess whether more stringent school meal nutrition standards would improve student weight status. Schools in states that exceeded USDA school meal nutrition standards with laws that encouraged a specified number of fruits and vegetables, reduced trans fats, low-fat or skim milk, and/or a minimum amount of whole grains had students with a more favorable weight status compared to schools in states with standards that did not exceed USDA standards. The study concluded that strict school lunch standards based on the latest nutritional standards could improve healthy eating habits and BMI among students who consume school lunches and especially those who participate in NSLP [13]. The relationship between policies around competitive foods and body weight was also examined prior to HHFKA. A cross-sectional study of 8th graders in one state demonstrated that school food practices that supported snacking and consumption of low-nutrient, energy-dense foods such as cookies, candy, and sweetened drinks was adversely associated with BMI [14].

Although the impact of the NSLP on child health has been studied, little is known about the predictors of participation in the school lunch program, particularly when healthier lunch entrees are introduced. The current study examined the association between BMI status and the purchase of school lunch meals in a low-income, racially diverse public middle school, when a year-long cafeteria intervention was in place to offer healthier alternative lunch meals alongside traditional school lunch meals. Individual students’ sociodemographic characteristics and their BMI data were linked to their cafeteria food purchases through the use of computerized point-of-sale cash registers. The purpose of this study was to determine whether a school-based environmental intervention effectively reaches its target audience: specifically, the students most vulnerable to childhood obesity risk. Of note, this study was conducted prior to implementation of the HHFKA. Thus, the modifications made to the school lunch meals were not mandated, but voluntarily undertaken by the school. By identifying predictors of school lunch participation in this way, this study has implications for future interventions and policies aimed at promoting healthy eating behaviors of middle school students.

## 2. Method

### 2.1. Intervention and Study Design

IMOVE is a school-based environmental intervention designed for middle school cafeterias to promote healthy eating behaviors through increased access to affordable, healthy, and portion-controlled school lunch meals that feature varying ethnic cuisines. Weekly menus are created to cater to the needs and cultural preferences of school-specific clientele, and are subsequently modified by consulting dietitians based on feedback from foodservice operators. Data for this study were obtained from an evaluation of IMOVE which assessed the cafeteria food purchases of students in an urban public middle school in Massachusetts during the 2008–2009 academic school year. 

This study was conducted prior to the implementation of HHFKA when standard school lunch meals adhered to the 1995 Dietary Guidelines for Americans. In contrast, IMOVE meals, which were served alongside the standard school lunch meals, met more stringent nutritional criteria by providing less than 25% of calories from total fat, less than 10% of calories from saturated fat, and more servings of fruits, vegetables, and whole grains. IMOVE offered a six-week cycle menu featuring recipes that cater to the needs and preferences of culturally diverse student populations. 

Monthly cafeteria events promoted the IMOVE program and incentivized students to increase and sustain healthy food choices and to encourage overall participation in school lunch. Events included fresh fruit and vegetable displays with free samples for taste-testing, raffle drawings and prize giveaways, IMOVE sticker logos to identify healthy choices at the point of selection, and attention-grabbing IMOVE posters. Students received a raffle ticket every time they purchased either an IMOVE hot meal or salad entree. Winners were chosen every 4 to 6 weeks to receive prizes that promoted physically active lifestyles (e.g., sports gear, backpacks, Frisbees). Twice yearly, a mountain bike was raffled.

### 2.2. Study Sample

Participants in this study: (1) were enrolled in the IMOVE intervention school during the intervention year; (2) had anthropometric data (height and weight) available to calculate BMI; and (3) had active status in the point-of-sale (POS) cash register system that tracked food purchases in the school cafeteria. Out of 477 students enrolled in the intervention school, 435 students had complete data and comprised the study sample.

Protocols, data collection instruments, and consent forms were approved by Boston University’s Institutional Review Board (October 1, 2007, IRB File #1678E). A strategy of passive informed consent with active parental dissent was approved given the non-invasive nature of the IMOVE program and the minimal risk to participants.

### 2.3. Instrumentation

The outcome variables were purchases made in the school cafeteria, obtained from a POS computerized cash register system that tracked daily cafeteria food and beverage purchases (Nutrikids™, LunchByte Systems, Rochester, NY, USA). The POS system created an electronic dataset that tracked purchases of all IMOVE meals, regular reimbursable school lunch meals, and á la carte foods sold on the lunch line.

These data were linked to the school district’s student enrollment data so that individual food purchases could be examined for students with varying sociodemographic characteristics. Enrollment data obtained from the school district identified each student attending the middle school by name, date of birth, sex, race/ethnicity, age, grade in school, and the student’s unique school ID. The enrollment database allowed for 62 different codes for race/ethnicity, which were combined into five categories based on the school’s demographics: Asian/Pacific Islander, Black/African American, Hispanic, White, and Multi-Race/Other Race. The enrollment data was the foundational database used to merge all of the other datasets as it contained both the 7-digit state ID that students used in the cafeteria cash register system and the 4-digit student ID used by school nurses and trained study staff when recording students’ height and weight for BMI computations. Each student, while wearing street clothes without shoes, was weighed and measured in a private location during the first three months of the school year by school nurses and trained study staff using standardized procedures.

### 2.4. Exposure Definition: Overweight Status

Sex-specific BMI-for-age percentiles were used to categorize the students as either obese (BMI-for-age ≥95th percentile), overweight (BMI-for-age 85th to <95th percentile), healthy weight (BMI-for-age 5th to 84th percentile), or underweight (<5th percentile) [15].

Some analyses used BMI as a binary variable that classified a child as overweight/obese (≥85th percentile) or not overweight/obese (<85th percentile) based on sex-specific BMI-for-age cutpoints [15]. These categorical definitions of overweight or obese are clinical determinations that schools report for the purposes of public health monitoring.

Other analyses used BMI as a continuous measure by employing BMI z-score, a standardized BMI that is calculated using an external reference population to statistically adjust for sex and age differences [16,17]. BMI z-score allows for increased interpretability over the crud, non-standardized BMI measure and is preferable to BMI-for-age percentile, which can be poorly suited for certain statistical analyses [18].

### 2.5. Outcome Definition: Purchase Rates for IMOVE and Standard School Lunch

The main outcome of interest was participation in IMOVE, which was assessed through school lunch purchases. Participation in IMOVE was expressed in terms of a *purchase rate*, which was defined as the total number of days on which a student purchased at least one IMOVE lunch divided by the total number of days on which the student made any type of cafeteria purchase (IMOVE lunch, standard lunch, or an á la carte item). Secondarily, participation in both standard school lunch and any school lunch was examined, using the same formula structure as noted for IMOVE participation. Thus, for IMOVE purchases, standard school lunch purchases, and any school lunch purchases, the denominator remained the same and the numerator reflected the total number of unique days on which at least one of the two types of lunch meals was purchased (IMOVE purchases and standard school lunch purchases) or either meal was purchased (any school lunch purchases). The number of unique days with any type of cafeteria purchase was chosen as the denominator in order to take into account both absenteeism and students bringing food to school. The distinction of “at least one” purchase on a given date was determined since students could purchase more than one school lunch or snack item per day. The derived purchase rate for school lunch was, therefore, a fraction that ranged from 0, meaning that a student never purchased the type of lunch in question (IMOVE lunch, standard lunch meal, or either type of lunch) on a day when he or she made a purchase in the school cafeteria, to 1, meaning that a student purchased that type of lunch every day that he or she made a purchase in the school cafeteria.

### 2.6. Statistical Analyses

Descriptive analyses were conducted to characterize the study population (*n* = 435) and the overall school sample (*n* = 477). For continuous variables, t-tests or non-parametric tests (Wilcoxon Rank Sum test or Kruskal-Wallis test) were used for stratified analyses as appropriate. Chi-square tests were used for categorical variables, or Fisher’s exact test when an expected cell frequency was ≤5. Correlation coefficients were calculated for pairs of continuous variables that appeared in scatter plots to be linearly associated.

Unadjusted analyses assessed the purchase rates for IMOVE by overweight/obese status and BMI z-score. The independent effect of each sociodemographic variable on IMOVE purchase rates was assessed using unadjusted regression modeling for categorical predictors or correlation analyses for continuous predictors. Purchase rates for standard school lunch and any school lunch were examined using the same methods.

Finally, adjusted regression models were developed to assess the effect of BMI on IMOVE purchase rates, controlling for age, sex, race/ethnicity, grade, and SES. Models were run for both categorical BMI status (overweight/obese *versus* not overweight/obese) and continuous BMI z-score. Parameter estimates with standard errors were reported to illustrate the estimated effect of overweight status and BMI z-score using analytic methods employed in similar studies [19]. All hypothesis tests were two-sided with a 0.05 level of significance. All analyses were performed with the SAS Statistical System version 9.1.3 [20].

## 3. Results

Students in this study had a mean age of 12.5 years. Students were evenly distributed among the three grades and by sex, with most students being either White (45.1%) or Asian/Pacific Islander (47.6%). African American and Hispanic students were underrepresented in this particular school. More than half of the students were eligible for free or reduced price lunch (54.5%) and were at a healthy weight (63.2%) (Table 1). Approximately 30% were above the 85th percentile for body weight, or about double the percentage in the reference sample used to determine the sex-specific BMI-for-age percentiles [15]. The study sample can be considered representative of the school’s entire student body as no significant differences were found between those included in this analysis and those excluded due to missing BMI data (data not shown).

Stratified analyses examined the sociodemographic variables by overweight status. The proportions of students in each weight category differed significantly by grade, with the highest proportion of overweight/obese students in 7th grade (41.1%) and the highest proportion of students with a healthy weight in 8th grade (38.6%, *p* = 0.03). There were significantly more males classified as overweight/obese (62.0%, *p* = 0.0005), and proportionately fewer Asian/Pacific Islander students in that category (*p* = 0.001) (Table 2).

**Table 1:** Study Sample Characteristics.

	Sample	
	**Study Sample (*n* = 435) ^1^ [% (n)]**	**Total School Sample (*n* = 477) ^1^ [% (n)]**
Age [mean (SD)]	12.5(1.0)	12.6 (0.9)
Eligible for free/reduced price lunch (SES indicator)	54.5 (237)	53.9 (257)
Body Mass Index (continuous)		
BMI [mean (SD)]	20.3 (4.8)	20.3 (4.8)
BMI z-score [mean (SD)]	0.3 (1.2)	0.3 (1.2)
BMI-for-age percentile [mean (SD)]	57.4 (32.9)	57.5 (32.4)
Body Mass Index (categorical)		
Underweight (<5th percentile)	7.1 (31)	7.1 (31)
Body Mass Index (categorical)		
Healthy Weight (5th–84th percentile)	63.2 (275)	63.2 (276)
Overweight (85th–94th percentile)	15.4 (67)	15.3 (67)
Obese (≥95th percentile)	14.3 (62)	14.4 (63)
Grade		
6th	31.5 (137)	30.6 (146)
7th	32.0 (139)	33.3 (159)
8th	36.6 (159)	36.1 (172)
Sex		
Male	49.2 (214)	49.6 (236)
Race/Ethnicity		
White	45.1 (196)	44.9 (213)
Black/African American	3.2 (14)	3.2 (15)
Asian/Pacific Islander	47.6 (207)	47.7 (226)
Hispanic	2.8 (12)	2.5 (12)
Multi-race/Other race/ethnicity	1.4 (6)	1.7 (8)

^1^ Sample sizes may be different for sociodemographic variables due to missing information on some participants.

**Table 2:** Sample Characteristics by Overweight Status, Study Sample (*n* = 435).

	Overweight/Obese (*n* = 129) [% (n)]	Not Overweight/Obese (*n* = 306) [% (n)]	*p*-value ^1^
Age [mean (SD)]	12.4 (0.9)	12.5 (1.0)	0.63
Eligible for free/reduced price lunch (SES indicator)	58.1 (75)	52.9 (162)	0.32
Grade			
6th	27.1 (35)	33.3 (102)	0.03
7th	41.1 (53)	28.1 (86)	
8th	31.8 (41)	38.6 (118)	
Sex			
Male	62.0 (80)	43.8 (134)	0.0005
Race/Ethnicity			
Asian/Pacific Islander	35.7 (46)	52.6 (161)	0.001
Black/African American	7.0 (9)	1.6 (5)	
Hispanic	4.7 (6)	2.0 (6)	
White	50.4 (65)	42.8 (131)	
Multi-race/Other race/ethnicity	2.3 (3)	1.0 (3)	

^1^ The Chi-square test was used for categorical variables; Fisher’s test was used for race/ethnicity. The t-test was used for continuous variables.

### 3.1. IMOVE and School Lunch Participation

Nearly all of the students eligible for the study made a purchase in the school lunch line during the intervention year (98.9%, *n* = 430). Unadjusted mean purchase rates for IMOVE lunch, standard school lunch, and any school lunch were calculated to compare students with varying sociodemographic characteristics (Table 3). Over the course of the school year, the mean participation rate was higher for the standard school lunch (mean = 0.77) than for the IMOVE lunch (mean = 0.72).

The main predictor of interest was BMI, which was assessed as both a dichotomous variable (overweight/obese *versus* not overweight/obese) and a continuous BMI z-score. BMI z-score was not found to be correlated with the purchase rates for IMOVE (correlation coefficient = 0.065, *p* = 0.18), standard lunch (correlation coefficient = 0.073, *p* = 0.13), or any school lunch (correlation coefficient=0.073, *p* = 0.13). However, some significant differences did emerge when BMI was examined as a categorical variable. Mean purchase rates were significantly greater for overweight/obese students than for those with a healthy weight for IMOVE (0.770 *versus* 0.700, *p* = 0.04), standard school lunch (0.821 *versus* 0.741, *p* = 0.05), and any school lunch (0.822 *versus* 0.742, *p* = 0.05) (Table 3).

Mean purchase rates for IMOVE lunch, standard lunch, and any school lunch were significantly associated with SES. Students who were eligible for free or reduced price lunch had higher mean purchase rates than those who were not eligible. Race/ethnicity was also significantly associated with purchase rates for both the IMOVE lunch and standard school lunch. Across all three outcomes, White students had the lowest mean purchase rates, while Asian/Pacific Islanders had the highest. Age was not significantly associated with mean purchase rates for any of the outcome variables. Grade was associated with IMOVE purchase rates, with 6th graders having the highest mean rate and 7th graders having the lowest. Though not significant, the same relationship was observed between grades and the purchase rates for both standard lunch and any school lunch, with 6th graders again having the highest mean purchase rates. Sex was significantly associated with mean purchase rates for IMOVE lunch, standard school lunch, and any school lunch, with higher mean purchase rates noted among boys (Table 3).

**Table 3:** Unadjusted Mean Purchase Rates for IMOVE Lunch, Standard School Lunch, and Any School Lunch by Participant Characteristics (*n* = 430).

	IMOVE Purchases [mean (SD)] ^1,2^	Standard Lunch Purchases [mean (SD)] ^1,2^	Any School Lunch Purchases [mean (SD)] ^1,2^
Age (correlation coefficient)	0.065	0.066	0.066
Eligibility for free/reduced price lunch (SES indicator)			
Eligible	0.852 (0.17) ****	0.905 (0.17) ****	0.905 (0.17) ****
Not eligible	0.556 (0.30) ****	0.595 (0.32) ****	0.596 (0.32) ****
Grade			
6th	0.744 (0.26) **	0.793 (0.28)	0.793 (0.28)
7th	0.669 (0.30) **	0.715 (0.32)	0.715 (0.32)
8th	0.738 (0.27) **	0.784 (0.28)	0.784 (0.28)
BMI z-score (correlation coefficient)	0.065	0.073	0.073
Body Mass Index (dichotomous)			
Not overweight/obese	0.700 (0.29) **	0.741 (0.31) *	0.741 (0.31) *
Overweight/obese	0.770 (0.23) **	0.821 (0.25) *	0.822 (0.25) *
Sex			
Male	0.786 (0.22) ***	0.842 (0.23) ****	0.842 (0.23) ****
Female	0.653 (0.31) ***	0.692 (0.33) ****	0.692 (0.33) ****
Race/Ethnicity			
Asian/Pacific Islander	0.838 (0.18) ****	0.892 (0.19) ****	0.892 (0.19) ****
Black/African American	0.806 (0.17) ****	0.869 (0.17) ****	0.869 (0.17) ****
Hispanic	0.786 (0.12) ****	0.839 (0.14) ****	0.839 (0.14) ****
White	0.578 (0.32) ****	0.615 (0.34) ****	0.615 (0.34) ****
Multi-race/Other race/ethnicity	0.723 (0.21) ****	0.772 (0.22) ****	0.772 (0.22) ****

^1^ *p*-values: * *p* = 0.05, ** *p* < 0.05, *** *p* < 0.001, **** *p* < 0.0001; ^2^ Spearman correlation was used for continuous predictors; non-parametric tests (Wilcoxon Rank Sum test or Kruskal-Wallis test) were used for categorical predictors to determine significance.

### 3.2. Adjusted Analyses

Multivariate adjusted regression analyses were performed to examine both dichotomous BMI (overweight status) and BMI z-score as predictors of participation in IMOVE lunch, standard school lunch, and any school lunch while controlling for age, sex, race/ethnicity, grade, and SES. Regression modeling estimated the effect of overweight status and BMI on purchase rates for IMOVE, standard school lunch, and any school lunch. 

Both overweight status and BMI z-score, when controlling for these sociodemographic variables, significantly predicted a student’s participation in IMOVE, standard school lunch, and any school lunch. Compared to students who were overweight or obese, those in the healthy weight range had 6.6% less participation in IMOVE, 6.8% less participation in standard school lunch, and 6.8% less participation in any school lunch. Additionally, with each unit increase in BMI z-score, participation in IMOVE increased by 0.026 days of IMOVE lunch purchases, participation in standard school lunch increased by 0.028 days of standard school lunch purchases, and participation in any school lunch increased by 0.028 days of any school lunch purchases (Table 4).

In these adjusted analyses, all of the sociodemographic variables examined, with the exception of age, were significantly associated with IMOVE, standard school lunch, and any school lunch participation, results that were similar to those observed in the unadjusted analyses. This was true whether BMI was measured by BMI z-score or examined as a dichotomous variable. Students who were eligible for free/reduced price lunch had roughly 0.21 higher mean participation in IMOVE, standard lunch, and any school lunch, meaning that they participated 21% more days than students who did not qualify for free/reduced price lunch (*p* < 0.0001). Male students had significantly greater participation than females in IMOVE, standard lunch, and any school lunch. White students had the lowest participation levels among the racial/ethnic groups, while 6th graders had the highest participation levels among the three grades. These associations were observed for both BMI models examined.

**Table 4:** Effect of BMI and Other Covariates on Participation Rates for IMOVE Lunch, Standard School Lunch, and Any School Lunch (*n* = 430).

	IMOVE Participation		Standard Lunch Participation		Any School Lunch Participation	
	Estimate [β (SE)] ^2^	*p*-value	Estimate [β (SE)] ^2^	*p*-value	Estimate [β (SE)] ^2^	*p*-value
****BMI Dichotomous (Overweight Status) Model ^1^****						
Overweight Status						
Not Overweight	−0.066 (0.024)	0.007	−0.068 (0.026)	0.008	−0.068 (0.026)	0.008
Overweight	-		-		-	
Age	0.010 (0.025)	0.69	0.011 (0.026)	0.67	0.011 (0.026)	0.67
Eligible for free/reduced price lunch (SES indicator)						
Yes	0.210 (0.025)	<0.0001	0.216 (0.026)	<0.0001	0.216 (0.026)	<0.0001
No	-		-		-	
Grade						
6th	0.045 (0.056)	0.02	0.052 (0.059)	0.02	0.052 (0.059)	0.02
7th	−0.042 (0.036)		−0.040 (0.038)		−0.040 (0.038)	
8th	-		-		-	
Sex						
Male	0.116 (0.022)	<0.0001	0.132 (0.023)	<0.0001	0.132 (0.023)	<0.0001
Female	-		-		-	
Race/Ethnicity						
White	−0.096 (0.091)	<0.0001	−0.105 (0.10)	<0.0001	−0.105 (0.10)	<0.0001
Black/African American	−0.020 (0.107)		−0.009 (0.113)		−0.009 (0.113)	
Asian/Pacific Islander	0.065 (0.091)		0.071 (0.096)		0.071 (0.096)	
Hispanic	0.040 (0.110)		0.046 (0.116)		0.046 (0.116)	
Multi-race/Other race/ethnicity	-		-		-	
****BMI Z-Score Model ^1^****						
BMI z-score	0.026 (0.009)	0.003	0.028 (0.009)	0.003	0.028 (0.009)	0.003
						
Age	0.010 (0.024)	0.69	0.011 (0.026)	0.67	0.011 (0.026)	0.67
Eligible for free/reduced price lunch (SES indicator)						
Yes	0.211 (0.025)	<0.0001	0.216 (0.026)	<0.0001	0.216 (0.026)	<0.0001
No	-		-		-	
Grade						
6th	0.050 (0.056)	0.01	0.057 (0.059)	0.01	0.057 (0.059)	0.01
7th	−0.041 (0.036)		−0.039 (0.038)		−0.039 (0.038)	
8th	-		-		-	
Sex						
Male	0.117 (0.021)	<0.0001	0.113 (0.023)	<0.0001	0.113 (0.023)	<0.0001
Female	-		-		-	
Race/Ethnicity						
White	−0.107 (0.091)	<0.0001	−0.117 (0.095)	<0.0001	−0.117 (0.095)	<0.0001
Black/African American	−0.021 (0.107)		−0.011 (0.113)		−0.011 (0.113)	
Asian/Pacific Islander	0.057 (0.091)		0.062 (0.10)		0.062 (0.10)	
Hispanic	0.025 (0.110)		0.030 (0.115)		0.030 (0.115)	
Multi-race/Other race/ethnicity	-		-		-	

^1^ Values are adjusted for the other variables in the model; ^2^ Values are estimated coefficients (with SE) for mean participation in school lunch, as calculated using linear regression modeling.

## 4. Discussion

To date, there has been little research on the association between the school food environment and children’s weight status [21]. Most of these studies have looked at BMI as a potential outcome of school meal participation rather than as a predictor of participation. Where it has been examined as an outcome, research has been inconclusive in relating school lunch participation to students’ BMI [1]. This study of middle school students is unique in its examination of BMI as a predictor of participation in both standard school lunch and a healthier, alternative school lunch offered alongside the standard meal. Participation was assessed objectively by monitoring lunch meal purchases in the school cafeteria. 

This study found that a student’s BMI was related to overall participation in school lunch, with heavier students exhibiting higher mean participation. Further, BMI also predicted the purchase of both the standard and alternative lunches. These associations were found whether BMI was measured by BMI z-score or examined as a dichotomous variable (overweight/obese *versus* not overweight/obese), even after adjusting for sociodemographic covariates. The results of this study also found other significant predictors of participation in school lunch, including non-White race/ethnicity, younger grade, being male, and having lower SES.

These results indicate that the students most in need will participate in school lunch when appropriate and appealing cafeteria meals are offered. Such interventions can be applied to a diverse body of students and can increase servings of fruit, vegetable, whole grain, and low-fat dairy. Non-white students from a variety of ethnicities participated regularly in IMOVE, suggesting that IMOVE meals were sufficiently varied and culturally acceptable. Males participated to a greater extent than females, as did students from low-income families.

These are important findings given the mixed reactions to the implementation of the HHFKA guidelines [6,7]. The HHFKA was enacted to update the nutritional requirements of school lunch, thereby changing the food offerings in school especially for those participating in government-sponsored meal programs. The findings suggest that appropriately planned cafeteria interventions to promote healthy eating do have the potential to impact students positively, including those who are most vulnerable to childhood obesity risks.

Mean participation rates were slightly higher for the standard school lunch meals than for the IMOVE meals. Nonetheless, these results indicated a high level of interest and participation in the IMOVE program among middle school students from a variety of sociodemographic groups. Most notably, participation in IMOVE was sustained throughout the school year and did not wane after a honeymoon period when the novelty of the new meals might have worn off. Sixth-graders participated in the IMOVE program the most, suggesting that efforts to appeal to younger students—for example, offering the incentive prizes—are worthwhile. An intervention like IMOVE that engages the youngest students and sustains their participation over time can help achieve the NSLP’s goals as the HHFKA guidelines are more widely adopted.

These findings have important implications for future interventions and policies designed to promote healthy eating behaviors among urban, low-income, and racially-diverse middle school students. First, it is clear that there are key predictors of school lunch participation for students, such as race/ethnicity and SES. Interventions should be planned and marketed to school districts with this in mind in order to achieve high levels of program participation among the students most likely to have poor eating habits and a greater risk of obesity. In addition, policy changes should consider the dietary preferences of different cultural groups with flexibility across schools to ensure the acceptability of school meal changes.

Second, as noted, this study showed that students who were overweight or obese had significantly higher mean participation in both standard school lunch and IMOVE. It is impossible from this study to tell whether being overweight/obese is a cause or consequence of school lunch participation, but the fact that IMOVE participation was similarly high among these students points to the possibility that improved school meals with better nutritional quality and portion control, if designed to appeal to students and creatively promoted, may positively affect their long-term eating habits and their health status.

There are two strengths of this study that deserve mention. First, school and research staff were trained to deliver the intervention to achieve implementation fidelity. Throughout the entire academic school year, research oversight was provided to the extent that budgetary constraints would allow. Second, the main outcome variables were objectively measured (cafeteria purchases) and not self-reported. Cashiers were trained prior to implementation on how to code food items in the POS system in order to minimize incorrect classification of the purchased foods.

It should be noted that the IMOVE program, as designed and delivered, was not comprehensive enough in scope to achieve broad and extensive improvements in children’s diets and obesity-related risks. For example, IMOVE did not modify or remove the standard school lunch entrees that had lower nutritional quality, higher energy density, or larger portions, nor did IMOVE address the quality, variety, or portion sizes of á la carte items or the foods available through school fundraisers. IMOVE did use point-of-purchase promotional signage and cafeteria events that allowed students to taste and learn about fresh fruits and vegetables, but did not offer a companion nutrition education curriculum for classroom use, nor did IMOVE have a physical activity component for improving time spent being physically active. Limited resources and issues related to profits, foodservice viability, limited kitchen equipment, space and skill level of foodservice staff, limited curriculum time, and low levels of administrative or community buy-in are substantial barriers to the kinds of multi-component intervention programs and large-scale policies that are typically required to have a more substantial impact on student behaviors and overall wellness. The HHFKA regulations provides guidance on how to address these and other limitations, but it remains to be seen whether this will result in schools adopting the kinds of multi-component intervention programs that are most likely to impact student behavior and health.

This study has three principal limitations. First, non-differential misclassification of the BMI exposure could have occurred if there were errors in the recorded height and weight measurements. This source of error was minimized by relying on school nurses and the trained study staff to take the measurements and by having duplicate entry of the data into the study database. 

Second, all participants in this study were from a single middle school in one urban public school district. Given that the school has an especially large proportion of Asian/Pacific-Islander students, the study’s findings should be cautiously generalized to other school settings. 

Finally, these findings may be influenced by reverse causation. Because only one BMI measurement was computed, it cannot be determined if students were overweight because they ate school lunch more regularly. That said, our analysis was a prospective investigation of school lunch purchase over one academic year, with BMI measured within the first one to three months of the school year. In spite of this limitation, these data underscore the opportunity to impact students by modifying the school lunch environment to provide healthier, portion-controlled offerings.

## 5. Conclusions

School-based environmental interventions, including policy changes, may positively impact child health and wellness by making healthy foods available and affordable, particularly to vulnerable populations. Schools are influential environments, and students in school can be reached at different levels of the socio-ecological model, including the environmental, interpersonal, and individual levels [14]. In particular, environmental level interventions can support a healthy lifestyle outside the school environment and reduce barriers that, in turn, can have a substantial impact on behaviors at the population level [22]. The IMOVE program is one such environmental strategy. This research identified BMI status as one of a few significant predictors of participation in a school-based cafeteria intervention. It also demonstrated that the school cafeteria is a viable intervention arena that has the ability to impact the nutritional status of the most vulnerable segments of the population in positive ways if modified and regulated with health promotion goals as a fixed priority.

The role that school foodservice has played in the childhood obesity epidemic has been of recent concern, and there is growing national attention on school meals and how the school food environment can be modified to improve children’s diets and weight [23]. To date, however, relatively few environmental-level interventions have been implemented, and even fewer have been rigorously evaluated to identify best practices for widespread replication. Results from the present study show that policies focused on healthy food intake should take into account not only how the environment influences students’ weight and behaviors, but how students’ current weight and behaviors might affect their participation in these interventions. In addition to further examining predictors of participation in both standard and alternative school lunch programs, future research that tracks weight status and relevant behaviors both before and after specific changes to the food school environment are implemented will help to determine which students the interventions are affecting and to what extent.
